# Perceptions of a Hospital’s Anesthesia Team Members on Precycling and Recycling of Anesthetic Gases

**DOI:** 10.3390/healthcare13030211

**Published:** 2025-01-21

**Authors:** Daniela Haluza, Katharina Brenn, Julia Choc, Julia Ortmann, Rafael Tschurtschenthaler, Lukas Schindler

**Affiliations:** 1 Department of Environmental Health, Center for Public Health, Medical University of Vienna, Kinderspitalgasse 15, 1090 Vienna, Austria; 2 Performance Center for Environmental Management and Hazardous Goods Consulting, VAMED-KMB Krankenhausmanagement und Betriebsführungsges.m.b.H, 1090 Vienna, Austria; 3 Department of Climate Neutrality and Transformation, Gesundheit Österreich GmbH, Stubenring 6, 1010 Vienna, Austria; 4 Department of General Surgery, General Hospital of Vienna, Medical University of Vienna, Währinger Gürtel 18–20, 1090 Vienna, Austria; 5 Department of Anesthesia, Intensive Care Medicine and Pain Medicine, Währinger Gürtel 18–20, 1090 Vienna, Austria

**Keywords:** green anesthesia, healthcare delivery, sustainability, sustainable development goals (SDGs), waste reduction, hospital management, charcoal adsorbers, desflurane, sevoflurane, inhaled anesthetics recapture, climate change

## Abstract

**Background:** Climate change poses significant challenges to global health, At the same time, the healthcare sector itself, with its high resource demands, also contributes substantially to global warming. Anesthesia, particularly through the use of volatile inhalation anesthetics, is a key contributor in this respect. The present exploratory study examines staff perceptions of precycling and recycling strategies aimed at reducing the environmental impact of anesthetic gases at the General Hospital Vienna, Austria. This large institution has recently implemented major changes, including the shutdown of the centralized nitrous oxide supply and the introduction of anesthetic gas recycling systems on anesthesia machines, alongside other precycling measures. **Methods:** We conducted a cross-sectional online survey of anesthesia team members (n = 103, 61.2% females) to assess current perceptions related to anesthetic gas usage, focusing on precycling and recycling aspects, and their willingness to engage in further sustainability efforts. **Results:** We found that participants expressed an, in general, positive attitude towards environmental protection measures and a high willingness to make additional efforts to recycle anesthetics. Thus, the anesthesia team members in our institution may be inclined to support strategies like minimizing the use of volatile anesthetics. **Conclusions:** These preliminary insights could inform actionable recommendations for advancing sustainable practices in anesthesiology at our hospital.

## 1. Introduction

Globally, climate change represents one of the greatest challenges for public health, with the healthcare sector itself causing significant greenhouse gas (GHG) emissions [1,2]. Anesthesia and intensive care medicine contribute significantly to this, particularly due to the use of volatile inhalation anesthetics [3]. Therefore, it is necessary to explore new ways to provide high-quality healthcare while acting sustainably. In Austria, like in all other countries, hospitals increasingly recognize that they can contribute to this endeavor [4]. A key area where hospitals see great potential to make a significant contribution to reducing GHG emissions is in the operating rooms [3]. Technical measures aimed at reducing energy consumption and utilizing gas-capture technologies, such as activated carbon adsorber systems, can limit the release of anesthetic gases into the atmosphere [4,5,6,7]. Although further evaluation of the full life-cycle impacts of recycling these captured gases is necessary, such technologies represent a promising step toward more sustainable anesthesia practices [7,8]. Within these, the field of anesthesiology is particularly relevant due to the use of climate-damaging anesthetic gases, given the power-consuming technical equipment and the huge amount of plastic and single-use equipment [9].

The two main methods for maintaining general anesthesia are propofol-based intravenous anesthesia and inhalation anesthesia with gases. Propofol, though commonly used, is highly toxic to aquatic environments and requires separate disposal via incineration to prevent contamination [10]. The inhalation anesthetics sevoflurane, desflurane, and nitrous oxide are potent GHGs that contribute to climate change. Nitrous oxide also contributes to the depletion of the ozone layer. Since these drugs are hardly metabolized by the body during anesthesia, over 95% of the inhaled amount is exhaled and, in most hospitals, released unfiltered into the atmosphere through the exhaust system. Desflurane has a much higher GHG potential than nitrous oxide and sevoflurane, although nitrous oxide remains in the atmosphere the longest.

From food waste to broken items and excessive packaging, the global waste output, particularly plastic waste, is projected to rise dramatically [11]. While governments are drafting regulations to reduce plastic use, consumers are adopting more sustainable consumption patterns. One such approach is “precycling”—a proactive strategy where individuals prevent waste before it accumulates by purchasing unpackaged goods, reusing items, or avoiding excessive packaging, as suggested by Leila Elgaaïed-Gambier [12]. Precycling aligns with the circular economy’s goal of zero waste by promoting sustainable, responsible consumption. Initially introduced to enhance the circular economy, precycling encourages consumers to adopt mindful behaviors that prioritize waste prevention [13]. Despite its growing relevance, precycling remains under-researched, particularly in understanding consumer motivations and behaviors that drive this practice [11].

In anesthesiology, the concept of precycling is gaining attention in the context of anesthetic gas usage [14]. Just as consumers can reduce their waste through precycling in daily life, healthcare professionals can apply similar principles to minimize the environmental impact of volatile anesthetics. Precycling in this context involves rethinking anesthetic practices to prevent waste before it occurs, whether through avoiding high fresh gas flow rates for all inhaled drugs, overall reducing the use of volatile gases, reusing equipment where feasible, or avoiding unnecessary emissions and waste [10]. By adopting precycling strategies in anesthesia, hospitals can reduce their GHG footprint and contribute to broader sustainability efforts, much like individuals who reduce plastic waste through conscious consumption. This innovative approach bridges the gap between environmental responsibility and clinical practice, fostering a more sustainable healthcare system [15].

In light of the growing awareness of environmental sustainability, this exploratory study at the General Hospital Vienna seeks to address these concerns by exploring innovative strategies to mitigate the environmental impact of anesthetic gases [16]. At the General Hospital Vienna anesthetic gases were previously released unfiltered into the environment through the exhaust air. To contribute to reducing General Hospital Vienna’s CO_2_ footprint, the Department of Anesthesia, General Intensive Care Medicine, and Pain Therapy decided to stop using nitrous oxide and test the anesthetic gas recycling system from the Germany-headquartered company ZeoSys Medical GmbH (Luckenwalde, Germany) [6]. This led to the following main research questions: What is the attitude of our anesthesia personnel towards measures for anesthetic gas recycling, and would our personnel be willing to accept additional effort?

## 2. Methods

### 2.1. Study Design

This cross-sectional exploratory study surveyed a non-random convenience sample of German-speaking employees at the General Hospital Vienna in Austria, who were part of the anesthesia team. For data collection, we utilized the online survey platform SoSci Survey [17]. This tool facilitated the design, distribution, and analysis of the questionnaire, allowing for secure and efficient data handling. The participants were primarily anesthesiologists and anesthesia nursing staff working at the university’s main teaching hospital, the General Hospital Vienna in Austria, which was part of the publicly funded university campus of the Medical University of Vienna. Participation in the survey was voluntary. All participants were informed about the purpose of this study and gave informed consent prior to participation. Data protection policies were strictly adhered to, ensuring both the anonymity and confidentiality of participants. To recruit survey participants, the questionnaire link and reminder messages were sent to approximately 250 employees within the anesthesia team at General Hospital Vienna, who constituted the target study population at the time of this study.

This study’s protocol was submitted for review to the Ethics Committee on 22 November 2023 and was approved on 19 January 2024. It was subsequently reviewed by the University’s Data Protection Committee, the General Staff Council, and the Scientific Staff Council at the Medical University of Vienna. These bodies imposed specific requirements for data processing and publication. According to their guidelines, gender designations must include the legally recognized categories: male, female, non-binary, intersex, open, or no response, data must be analyzed in accordance with Good Scientific Practice Publication of results from groups with fewer than seven participants was prohibited. Data usage was restricted to the purpose stated in the application, and data analysis must ensure that individuals could not be identified, including through open-ended responses. The survey was conducted following the principles of the Helsinki Declaration and in compliance with the EU General Data Protection Regulation.

### 2.2. Study Questionnaire

The questionnaire collected general information, including age, gender, main residence, professional activity, and length of service. Another set of questions focused on participants’ self-assessment of their interest in environmental and climate protection topics, their level of information and concern about these issues, their assignment of responsibility, and their action-oriented attitudes and personal activities related to environmental and climate protection. The central section of the questionnaire gathered information on the use of anesthetics in the hospital, personal experience with recycling volatile anesthetics, and personal attitudes toward recycling these substances. Additionally, an open-ended question, “Would you like to tell us anything else?” provided space for survey participants to offer further comments in a text field.

The questions and the overall structure of the questionnaire were tested in a qualitative-semantic pretest (n = 23), and the questionnaire was modified based on the test results. The pretest was conducted online using the Sosci Survey pretest function between 9 and 23 October 2023 [17]. A total of 23 participants took part in the pretest (mean 38.4 years, SD 11.5, age range from 18 to 57 years). Most participants were female (n = 14, 60.9%), while eight participants (34.8%) were male, and one participant (4.3%) identified as diverse. The test subjects received the online link to the questionnaire and were asked to answer the questions to check the flow and understandability of the survey and provide feedback on the clarity of the questions, the order of the questions, and possible misunderstandings. Based on this feedback, necessary adjustments and improvements were made to the questionnaire. The pretest ensured that the survey was understandable for the actual data collection and helped identify potential errors or misunderstandings in the questions in advance, thereby improving the quality of the collected data.

### 2.3. Statistical Data Analysis

The statistical analysis of the survey data was conducted using SPSS Statistics for Windows (Version 29.0, IBM Corp., Armonk, NY, USA). To construct variables, mean scores were calculated from the scales measuring interest in environmental and climate protection topics. Descriptive statistics were employed to present the quantitative results, including percentages, means (M), and standard deviations (SD). For improved interpretability, items in the tables were sorted in descending order by mean score. Means were computed from the four response options ranging from “strongly disagree” to “strongly agree”, excluding neutral responses (“don’t know/no response”). However, these neutral responses were included in frequency calculations. To facilitate the interpretation of frequencies, a dichotomized variable was created for the question block on attitudes towards anesthetic gas recycling, classifying responses into “disagree” versus “agree”. For median splitting in the age variable (younger than 37 years and 37 years and older), the median was calculated. For qualitative content analysis, free-text responses were reviewed and coded to extract information from unstructured text data and derive insights [18]). To analyze subgroup differences in response patterns, a dichotomized variable was created for free-text responses (yes or no).

## 3. Results

### 3.1. General Sample Characteristics

Out of the about 250 employees in the anesthesia team of the General Hospital Vienna, a total of 192 clicks on the questionnaire were recorded, including accidental double clicks. Of these, 105 participants provided complete data, resulting in a 54.7% completion rate among those who started the survey. Data collection took place between 6 February and 26 March 2024. The average completion time was 4.5 min (SD 1.0, range between 2.4 and 7.2 min). Notably, the inclusion criteria for the survey were adults who agreed to participate in the survey (consenting to the consent form at the beginning of the survey), were employed at the General Hospital, and were able to complete an online questionnaire in German. Two data sets were subsequently excluded because the individuals indicated they did not work at the General Hospital, resulting in a total study population of n = 103.

The first section collected general information on age, gender, primary residence (rural or urban area), job type in the anesthesia team (anesthesiologist, resident in anesthesiology, nursing staff, operation room staff), and work experience as measured in length of service (up to 5, 5–10, more than 10 years). On average, the participants were 40.1 years old (SD 10.9 years, age range 22 to 64 years, median 37 years). Using median splitting, participants were divided into two groups with lower age (n = 53, 51.5%) and higher age (n = 50, 48.5%). The majority of participants (61.2%) were females and 81.6% lived in urban areas. Additionally, 40.8% reported being part of the operating room and nursing staff, and 51.5% had more than 10 years of work experience. The last two variables were also dichotomized, showing a balanced percentage of medical and non-medical staff as well as lower and higher service duration (Table 1). One participant selected ’diverse/inter/open/no answer’ in the gender category. Due to the single response, gender was dichotomized into two categories (female and male) for the gender analysis. However, the non-binary participant’s data were retained in the overall data set to ensure completeness and inclusion of all responses.

### 3.2. Interest in Environmental and Climate Protection

The third section of the online survey gathered data on topics related to environmental and climate protection. This section focused on participants’ levels of interest in specific environmental and climate protection issues, as well as their personal actions and attitudes toward these topics. The response patterns indicated minimal neutrality among participants, with a neutral response rate of less than 2.0%. Table 2 shows the results of the descriptive statistics. When comparing the levels of agreement across the different areas, ’interest in environmental and climate protection actions’ ranked lowest (mean = 2.8, SD = 1.0). In contrast, the highest levels of agreement were observed for ’energy saving at home’ and ’protection of rare animals, plants, and habitats’ (mean = 3.7 each, maximum score of 4). The remaining areas fell between these two extremes. Notably, neutral responses such as “I don’t know/no answer” were excluded from the calculation of the mean scores to ensure clarity in interpreting participants’ genuine interest levels.

The highest average agreement was observed on average for the statements “Our descendants will suffer from the consequences of environmental pollution” and “The economy should make a much greater contribution to environmental and climate protection” (both M = 3.8, SD = 0.5; see Table 3). In contrast, the lowest average agreement was found on average for the statements “I, as an individual, cannot make a significant contribution to environmental and climate protection” (M = 2.5, SD = 1.0) and “I don’t know what I could do for environmental and climate protection” (M = 1.8, SD = 0.8).

### 3.3. Attitudes Toward Recycling Anesthetics

The survey explored current practices and perceptions regarding sustainable anesthesia in the hospital setting. At the time of data collection, the adsorber system, i.e., the charcoal-based system for capturing anesthetic gases from Zeosys, was only used in 3 out of 60 operating rooms as part of a test phase, and the nursing staff was rarely assigned to surgeries outside their specialty unlike the medical staff [6]. The rollout to additional operating rooms is already planned. Table 4 shows the frequencies of the response options regarding the practices of anesthetic use at the General Hospital Vienna, as well as their average values, the latter excluding the neutral response option.

The practice of low-flow anesthesia was highly reported, with a mean score of 3.6 (SD = 0.6), indicating frequent use by most respondents; 68% indicated that low-flow anesthesia was regularly or always applied to reduce volatile anesthetic consumption. Automated regulation of fresh gas flow and volatile anesthetic dosing showed a lower mean score of 1.9 (SD = 1.1), with only 21% of respondents reporting regular or frequent use.

As for the use of the Zeosys adsorber system to capture and recycle anesthetic gases, respondents reported a moderate average score of 2.7 (SD = 1.0). Around 40% of respondents reported some level of use, though 34% indicated infrequent or no implementation. In terms of specific anesthetic agents, the avoidance of desflurane—recognized for its environmental impact—was reported with a mean of 2.9 (SD = 1.1). Approximately 45% of respondents reported often or always avoiding desflurane, while the remaining responses indicated inconsistent avoidance. Lastly, the presence of a “green team” to support sustainability efforts was prominent, with a mean score of 3.7 (SD = 0.7). More than 80% of respondents acknowledged regular or consistent efforts by such teams, suggesting that institutional support for environmentally friendly practices was generally strong.

The analysis of personal attitudes towards the recycling of volatile anesthetics showed a significant number of neutral responses, particularly for the statement regarding cost savings in healthcare through recycling. More than a quarter of respondents selected “don’t know” or gave no answer for this item, while around 10% chose the same response for other items in the question block. To simplify interpretation, the data were dichotomized disagree vs. agree, along with the neutral response), which provided clearer insights into overall attitudes (Table 5).

The majority of participants expressed support for recycling anesthetics, with 91% willing to accept the additional effort involved. A large proportion, 88%, agreed that recycling is beneficial for environmental and climate protection and considered recycled anesthetics safe from a health perspective. Additionally, about two-thirds of respondents believed that recycling would save costs in healthcare and expected that it would soon become standard practice at their hospital. Lastly, nearly 60% of participants indicated that they were knowledgeable about the recycling of volatile anesthetics, reflecting a general awareness of the issue among the respondents.

### 3.4. Free-Text Analysis

Qualitative feedback was gathered through an open-ended question, with only 15 participants (14.6%) voluntarily providing responses, constituting a small fraction of the total sample. These comments were analyzed using qualitative content analysis, employing an inductive, data-driven approach to identify three main themes: sustainable practices, waste management, and general comments. Table 6 presents the free-text responses with subgroup comparisons based on demographic and professional factors. A slightly higher number of younger participants (n = 8) compared to older participants (n = 7) contributed comments. The responses were predominantly from women (n = 11) and medical doctors (n = 10), with fewer men (n = 4) and non-doctoral staff (n = 5) contributing.

## 4. Discussion

The growing concern over the environmental impact of processes has led to an increasing body of literature addressing the necessity for sustainable practices in healthcare, particularly in anesthesia [3,9,10,19]. In addition to precycling strategies, such as minimizing fresh gas flow rates, anesthetic gas recycling technologies are already available to reduce greenhouse gas (GHG) emissions without compromising patient safety or care quality [7,14]. We surveyed the anesthesia team at the General Hospital Vienna to explore their attitudes toward environmental measures, focusing on their support for anesthetic gas precycling and recycling, including the feasibility of adopting ZeoSys Medical GmbH’s novel recycling system [6].

Our findings revealed strong support for sustainability initiatives, with over 80% of respondents willing to adopt anesthetic gas recycling. However, nearly 60% indicated limited knowledge about recycling volatile anesthetics, underscoring the need for targeted education and training to improve awareness and facilitate implementation [19]. Additionally, we explored interest in environmental and climate protection topics. The results suggest that respondents are primarily motivated by personal, actionable steps, but show less enthusiasm for broader systemic or collective climate protection efforts [20]. Respondents reported a high engagement with household energy conservation and protecting rare species and habitats—actions that are usually immediate and accessible in daily life [21]. In addition, we found notable concerns about environmental pollutants and a preference for sustainable products, reflecting mindfulness of how consumption affects health and the environment. In contrast, engagement was lower when it comes to climate change and climate protection, which may seem broader, more abstract, and harder to influence individually [22].

The qualitative content analysis of open-ended responses utilized a data-driven, inductive approach to categorize and interpret themes relevant to the research questions [18]. Despite a limited number of comments from a small subset of the most motivated respondents (n = 15), which may not be representative of the target population or the broader respondent pool, the feedback indicated support for sustainability initiatives at the General Hospital Vienna, and these preliminary insights may not be generalizable to other institutions. However, concerns were raised regarding waste segregation in operating rooms and the need for additional staff training. Several key points emerge from the comments: the energy-intensive nature of these medical fields, the necessity for hospitals to embrace sustainable innovations, and the perception that volatile anesthetics can be replaced with minimal clinical consequences. These findings point to the necessity of further efforts to effectively implement and integrate eco-friendly practices throughout the hospital [23].

Under the theme of sustainable practices, comments reflect an awareness of the energy-intensive nature of anesthesia and interest in innovations like renewable energy solutions and green mobility. One respondent highlighted, “Volatile anesthetics can largely be replaced without negative effects!” (male, medical doctor), which aligns with existing literature on reducing the environmental impact of anesthetics [1,2,4,20,23,24]. These perspectives suggest that the respondents were open to adopting more sustainable practices, although the broader hospital population may not share this enthusiasm due to the limited exposure to these options. In the waste management category, respondents pointed out inefficiencies in current practices, such as unused equipment and improper waste separation. As one comment noted, “Waste separation in the OR still doesn’t work; no one follows the guidelines. Waste separation training for all staff is needed”. (female, non-doctoral staff, with OR meaning operation room). This highlights the gap in training and compliance, which is consistent with research emphasizing the need for improved waste management protocols [15].

The general comments category contained responses that appreciated the sustainability initiatives but also acknowledged their limited impact in the broader context. As one respondent stated, “I wish there were more information and engagement from all employees in this direction! Thank you!” (female, non-doctoral staff). This suggests that while there is support for sustainability efforts, greater engagement and education across departments are necessary to create a culture of sustainability [19,25]. Overall, while these insights are valuable, they represent the opinions of a small portion of respondents, and further research is needed to assess the broader applicability of these attitudes and identify strategies to overcome barriers to sustainable practice adoption in our institution [4]. To effectively integrate sustainability into healthcare practices, it is crucial to develop and establish sustainable management methods that enhance motivation for environmental protection among employees. Evidence suggests that employee engagement is a key driver of successful sustainability initiatives in healthcare settings [26]. Training and awareness-raising initiatives can educate staff on the importance of environmental protection, helping them understand the direct impact of their actions on the environment and the positive outcomes their efforts can achieve [1].

While this exploratory study revealed high interest from anesthesia personnel, reflected in a response rate of over 50%, it is important to note that few respondents reported using technology to support environmentally efficient anesthetic agent delivery. This was primarily due to the limited availability of such technologies in our hospital, rather than a lack of practitioner involvement. As this area is still in its early stages at our institution, further research is needed to explore the barriers to technology adoption and how increased access to these tools could promote more sustainable practices [25,26]. Understanding the full impact of precycling and recycling on anesthetic gas emissions will require additional studies to provide deeper insights into the challenges and opportunities for practice improvement in our hospital. Notably, proactive support from hospital management is critical in promoting sustainable practices [27]. Healthcare organizations should motivate staff to adopt environmentally friendly practices, contributing to environmental protection [28]. Given the usefulness of precycling and recycling attempts, understanding anesthesia team members’ attitudes and practices is crucial for developing effective GHG reduction strategies [13,27]. Moreover, this research fills a significant gap in the literature by collecting the perspectives of those directly involved in anesthetic practices [14]. However, further research is needed to evaluate the long-term effectiveness and feasibility of these approaches in clinical settings [7,10,15].

The aim of our exploratory study was to provide an exploration of attitudes toward anesthetic gas usage within the unique setting of the General Hospital Vienna. Given the size of our hospital, understanding the perceptions of its staff, in general, and anesthesia team members specifically provided preliminary insights that may guide similar initiatives in other large hospitals. As healthcare systems globally strive for greater sustainability, this research provides a timely and necessary contribution to the dialog surrounding waste management in anesthesia [24].

As for the practical implications of this study’s findings, staff enthusiasm for sustainable strategies highlights opportunities to reduce the environmental impact of anesthetic practices, but implementation is hindered by practical barriers. Limited access to low-flow anesthesia technology and minimal experience with anesthetic gas recycling systems constrain adoption. While recycling holds promise, its current impact is negligible due to technological and infrastructural challenges. Precycling, on the other hand, appears more feasible in our setting. Advancing these practices requires increasing access to low-flow equipment, enhancing staff training, and addressing institutional barriers. These insights can guide hospitals toward more sustainable anesthetic practices while underscoring the importance of employing multivariate methods and using larger, more diverse samples to enhance the generalizability of findings [1,23,24,29].

### Limitations

This exploratory study used an online survey to gather perceptions from anesthesia team members at the General Hospital Vienna, with some limitations. Primarily, online surveys are prone to selection bias, attracting more motivated, environmentally conscious respondents, and limiting generalizability to the entire workforce. While recycling technologies were explored, they were not widely implemented at our hospital, with precycling efforts being more feasible. Future research should include a more representative sample to better assess sustainable practices across healthcare disciplines. Secondly, the self-reported nature of the data introduces social desirability bias, where respondents may over-report behaviors perceived as desirable [30]. This bias may lead to an overestimation of environmental awareness and sustainable practices among participants. Additionally, the cross-sectional design captures only a single point in time, which limits the ability to track changes in environmental attitudes or practices over time [2]. Longitudinal studies would be beneficial to assess how these attitudes evolve in response to interventions or over time.

While free-text responses can provide rich, qualitative insights, they are also subject to interpretative bias during coding and analysis [18]. Variability in how different researchers interpret and categorize responses can affect the consistency and reliability of the findings [31]. Finally, this exploratory study’s focus on a single institution may restrict the applicability of findings to other healthcare settings with different organizational cultures or resources. Forthcoming research should aim to include multiple institutions to enhance the generalizability of the results and to explore how different contexts influence sustainable practices in anesthesiology [32]. By acknowledging these limitations, these findings provide a foundation for more comprehensive research that can better inform strategies for enhancing sustainability in anesthesiology and contribute to climate-neutral hospital operations—achieving net-zero GHG emissions through a combination of emissions reduction and offsetting measures.

## 5. Conclusions

This exploratory study of our hospital’s anesthesia personnel bridges theory and practice by highlighting the critical role of staff involvement in developing effective strategies for recycling and precycling anesthetic gases. As the medical community becomes more aware of sustainability challenges, exploring innovative solutions to mitigate waste and enhance anesthetic practices is crucial. Recycling and precycling play key roles in this effort. Recycling recovers and reuses anesthetic gases while precycling focuses on preventing waste at the source by optimizing usage and reducing gas flow. While precycling is crucial for waste prevention, this survey offers preliminary insights into recycling adoption, influenced by limited access to recycling technology and respondents’ general environmental predisposition, rather than direct experience with the practice. Future research should further explore the barriers to precycling and recycling adoption, focusing on the role of technology, training, and institutional support in overcoming these challenges. Such research will be crucial for understanding how to translate positive attitudes into practical, sustainable changes that reduce anesthetic gas emissions in real-world clinical settings.

## Figures and Tables

**Table 1:** Sociodemographic characteristics of the study population (n = 103).

Item	N	%
Gender		
Female	63	61.2
Male	39	37.9
Diverse	1	1.0
Primary residence		
Rural area	19	18.4
Urban area	84	81.6
Profession		
Anesthesiologist	29	28.2
Resident in anesthesiology	24	23.3
Nursing staff	42	40.8
Operation room staff	8	7.8
Profession (dichotomous)		
Medical doctor	53	51.5
Non-doctoral staff	50	48.6
Length of service		
Up to 5 years	29	28.2
5–10 years	21	20.4
More than 10 years	53	51.5
Length of service (dichotomous)		
Lower (up to 10 years)	50	48.5
Higher (more than 10 years)	53	51.5

**Table 2:** Interest in environmental and climate protection topics, sorted ascending by mean.

Item	Mean	SD
Saving energy in the household	3.7	0.5
Protection of rare animals, plants, and habitats	3.7	0.6
Pollutants in the environment	3.6	0.6
Sustainable, environmentally friendly products	3.5	0.6
Healthy products	3.5	0.6
Climate-friendly mobility	3.5	0.6
Climate change and climate protection	3.3	0.8
Environment and climate protection campaigns	2.8	1.0

**Table 3:** Information status and current worries concerning environmental and climate protection, sorted ascending by mean.

Items	Mean	SD
Our descendants will suffer from the effects of environmental pollution.	3.8	0.5
The economy should make a much stronger contribution to environmental and climate protection.	3.8	0.5
I believe that environmental and climate protection is the responsibility of all citizens.	3.6	0.6
I personally feel responsible for preserving nature.	3.5	0.7
People have lost control over their impact on the environment.	3.4	0.7
To protect nature, many more citizens would need to commit to environmental and climate protection.	3.4	0.8
I actively contribute to environmental and climate protection.	3.2	0.7
I feel well-informed about the topic of environmental and climate protection overall.	3.1	0.7
The more information I receive about environmental pollution, the more uneasy I feel.	3.1	0.9
I am afraid for the future when I think about our environment.	3.0	0.9
The government is primarily responsible for environmental and climate protection.	2.8	0.9
As an individual, I cannot make a significant contribution to environmental and climate protection.	2.5	1.0
I do not know what I could do for environmental and climate protection.	1.8	0.8

**Table 4:** Information on anesthetic use practices at the General Hospital Vienna.

Items	Strongly Disagree		Disagree		Somewhat Agree		Agree		Neutral		Mean	SD
In My Hospital…	n	%	n	%	n	%	n	%	n	%		
…the use of low-flow anesthesia to reduce the consumption of volatile anesthetics is practiced.												
	0	0	6	5.8	27	26.2	56	54.4	14	13.6	3.6	0.6
…an automatic regulation of the fresh gas flow and the dosing of the volatile anesthetics is used.												
	39	37.9	23	22.3	10	9.7	12	11.7	19	18.4	1.9	1.1
…special adsorber systems are used to capture and recycle anesthetic gases.												
	13	12.6	22	21.4	26	25.2	23	22.3	19	18.4	2.7	1.0
…desflurane is avoided as an inhalation anesthetic.												
	12	11.7	20	19.4	24	23.3	31	30.1	16	15.5	2.9	1.1
…there is a ’green team’ to promote sustainability in healthcare facilities.												
	3	2.9	4	3.9	12	11.7	63	61.2	21	20.4	3.7	0.7

**Table 5:** Personal attitude towards recycling volatile anesthetics (frequency). To better interpret the results, the dichotomized variables (disagree vs. agree, along with the neutral response) were created.

Items	Neutral		Disagree		Agree	
	n	%	n	%	n	%
I already have experience and knowledge about the recycling of volatile anesthetics.	11	10.7	31	30.1	61	59.2
Recycling anesthetics is beneficial for environmental and climate protection.	12	11.7	0	0.0	91	88.3
Recycling anesthetics saves costs in the healthcare sector.	29	28.2	4	3.9	70	68.0
From a health perspective, recycled anesthetics are safe.	12	11.7	0	0.0	91	88.3
I accept the additional effort required to recycle anesthetics.	8	7.8	1	1.0	94	91.3
I believe that recycling of volatile anesthetics will soon be the standard in my hospital.	10	9.7	36	35.0	67	65.0

**Table 6:** Free-text responses (n = 15), stratified by the dichotomous variables age category, gender, and profession.

Number	Free Text	Age Group	Gender	Profession
I.	Sustainable practices			
1	Anesthesia/intensive care is a very energy-intensive field (especially compared to others like psychiatry, etc.). Hospitals should establish innovations for sustainable energy generation (e.g., solar panels on parking lot roofs, wind turbines at the hospital, greening of facades, …).	Younger	Male	Medical doctor
2	I would like my employer to promote “green mobility”, such as offering annual public transportation passes for those who do not use a garage space, or providing secure bike parking.	Younger	Female	Medical doctor
3	Volatile anesthetics can largely be replaced without negative effects!	Older	Male	Medical doctor
4	It would be desirable if surgery (nurses and doctors) would get involved in the sustainability issue.	Older	Female	Non-doctoral staff
5	I believe that the people from my department who are participating in this survey are those who already find sustainability important. Thus, I believe the survey is subject to strong selection bias. Nevertheless, I think it is great that you are addressing this issue, and I am already trying to make my work and life as environmentally friendly as possible.	Younger	Female	Medical doctor
II.	Waste management			Medical doctor
6	Waste reduction and control. Infusions are left behind after surgery. New arterial sets are opened in the ICU, and ventilation tubes and accessories are not used according to manufacturer recommendations. A breathing tube with a filter can be used for up to a week. A monitor can be used for over 48 h, and there are many more examples.	Older	Female	Medical doctor
7	Waste separation in the OR still does not work; no one follows the guidelines. Waste separation training for all staff is needed.	Younger	Female	Non-doctoral staff
III.	General comments			
8	Nuclear power, no thanks.	Younger	Female	Non-doctoral staff
9	Thank you for your initiative.	Older	Female	Non-doctoral staff
10	Thank you for your efforts!	Older	Female	Medical doctor
11	I think these actions are good and important. However, we are dealing with about 0.1% impact. I know everyone should start with themselves first, but we shouldn’t lose sight of the “big picture”!	Older	Male	Medical doctor
12	I mostly perform TIVAs (Total Intravenous Anesthesia).	Younger	Female	Medical doctor
13	I wish there were more information and engagement from all employees in this direction! Thank you!	Older	Female	Non-doctoral staff
14	Great initiatives!	Younger	Male	Medical doctor
15	Thank you for your continuous work and dedication.	Younger	Female	Medical doctor

## Data Availability

The data presented in this study are available on request from the corresponding author. The data are not publicly available due to institutional restrictions.
